# Supplementary material for: The Need for Culturally Appropriate Food Allergy Management Strategies: The Indian Milk Ladder
Source: Nutrients. 2023 Sep 9;15(18):3921. doi: 10.3390/nu15183921 (PMC10535525; doi:10.3390/nu15183921)
Supplement: Supplementary file 1 [file nutrients-15-03921-s001.zip › nutrients-2565723-supplementary.pdf]

Supplementary Information S1: Detailed Recipes

MAIDA DIAMOND BISCUIT						
**Recipe**	**tsp/tbsp/ cup**	**g/ml**	**oz/lb**	**Ingredient**	**Cooked Milk protein per 100 grams**	**Temperature**
Maida Diamond biscuit					0.006 grams	
**Bakes 40 small finger size biscuits**<br>**Sugar:0.75 grams/biscuit**	1 cup	240 g	8.46 oz	Flour (maida/all-purpose flour)		Pre-heat the oven to 180c for 10 mins, then bake the biscuits for 15 minutes.
	¼ cup	60 g	2.11oz	Powdered sugar		
		50 ml	1.69oz	milk		
	2 tbsp	30gms	1.05oz	ghee/butter		
	1 big pinch			Salt and Baking soda		
	Tip of a knife			cardamom powder		

1. Grind the sugar and the cardamom in a small mixie / grinder to a smooth powder. Set aside

2. Mix the Maida (All-purpose flour), sugar mixture, a big pinch of salt and a big pinch of baking soda. Add 2 tablespoon of ghee/butter & 50ml milk. Combine well with fingers to make sure the ghee/Butter is evenly mixed

3. Start adding water, add very little at a time. Make a very stiff dough. Make balls out of the dough. Set aside. Dust the dough ball in maida generously and roll each dough ball into a flat circle. Don't roll it too thin. Roll it a little thicker than chapati

4. Cut the circle into little diamonds with the help of a pie cutter or a knife

5. Heat oil in a pan in medium flame. Gently add the diamond cuts into the oil. Fry the biscuits until golden colour is obtained

For baked version - Pre-heat the oven to 180c for 10 mins, then bake the biscuits for 15 minutes

<table>
<tr><th colspan="7">MILK COOKIE</th></tr>
<tr><th>Recipe</th><th>tsp/tbsp/ cup</th><th>g/ml</th><th>oz/lb</th><th>Ingredient</th><th>Cooked Milk protein per 100 grams</th><th>Temperature</th></tr>
<tr><td colspan="5"></td><td>0.36 grams</td><td></td></tr>
<tr><td rowspan="10">Bakes 16 milk cookies

Sugar: 1.9 grams/cookie</td><td>1 ½ cups</td><td>360 g</td><td>12.69 oz</td><td>All-purpose flour</td><td rowspan="10"></td><td rowspan="10">180-degree Centigrade

Baking time-10-12mins

Tip: Once done, immediately transfer the cookie sheet from the hot oven, to prevent over baking.</td></tr>
<tr><td>¾ cup</td><td>30</td><td>6.3oz</td><td>Sugar</td></tr>
<tr><td>1 tsp</td><td>5 g</td><td>0.17oz</td><td>Baking powder</td></tr>
<tr><td>2 tbsp</td><td>30 g</td><td>1.05oz</td><td>Dry milk powder</td></tr>
<tr><td>½ tsp</td><td>2.5gms</td><td></td><td>Salt</td></tr>
<tr><td>½ cup</td><td>120ml</td><td>4.23oz</td><td>Butter</td></tr>
<tr><td></td><td></td><td></td><td></td></tr>
<tr><td>½ cup</td><td>120ml</td><td>4.23oz</td><td>Water</td></tr>
<tr><td colspan="4">Vanilla essence to taste</td></tr>
</table>

Step -I: To make the dough

1.Mix all the dry ingredients evenly – flour, baking powder, salt and milk powder

2.Add melted butter, sugar in another bowl, then add water and vanilla extract to the butter sugar mixture and stir it well

3.Add the wet ingredients mixture slowly to the dry flour mixture to get a crumbly mixture. Knead well

4.Transfer the bowl with cookie dough to the refrigerator and chill in the refrigerator for about 15 to 20 minutes (do not chill for more time as the dough may harden)

Step II: To make the cookies

5.Make small balls out of the dough and then slightly press the balls to form the round cookie shape

6.Place this cookie in the baking sheet. Similarly shape all round cookies out of the dough and place them all in the cookie sheet

Step III. To bake the cookies

7.Preheat oven to 350-degree Fahrenheit

8.Place the cookie sheet in the preheated oven and bake for about 10 to 12 minutes

GAJAR HALWA

Recipe	tsp/tbsp/ cup	g/ml	oz/lb	Ingredient	Cooked Milk protein per 100 grams	Temperature
					0.41 grams	
	2 cups	500ml	17.6 oz	Milk		
	¼ cup	500 g	1.76 oz	Carrot		
	2 TBSP	30g	1.1 oz	Ghee		Cook in a hot pan until desired consistency is achieved.
	½ cup	120 g	4.23	Sugar		
**Serving size – 20**	2 tbsp	30g	1.1 oz	Nuts &Dry fruits(optional)		(Boiled: 25 minutes)
**Sugar 6 grams/serving**	Tip of knife			Cardamom powder		

STEPS

1. Add Grated carrot and Milk in a pan.
2. Cook on a low flame by stirring continuously until soft and milk reduces to 75%.
3. Add Ghee and mix well

4. Add required sugar and cardamom powder and cook on low flame

5. Lastly add nuts and dried fruits (if required) and cook until the mixture looks dry with milk solids appearing in the halwa

6. It’s fine even if the halwa has some moisture

GULAB JAMUN (DRY)						
**Recipe**	**tsp/tbsp/ cup**	**g/ml**	**oz/lb**	**Ingredient**	Cooked Milk Protein Content per 100 grams	**Temperature**
					1.23 grams	
"Instant Ready mix is available" – (100 gm makes about 20 Gulab Jamuns)<br><br>Sugar: 5 grams/jamun	7 Tbsp	100g	3.5oz	Instant Ready-mix ingredient		Fry in a hot pan using oil of choice until golden brown and crispy.<br><br>(Boiled+ fry: 20+5 minutes)
				Refined wheat flour		
				Milk solids		
	½ cup	100gms	3.5 oz	Sugar		
	½ cup	100 ml		Water (for sugar syrup)		
				Oil (for deep frying)		
1.Add required water gradually to the ready-mix powder and knead to make a smooth dough. Keep aside for 5 min<br><br>2.Apply oil/ghee on both palms and shape the dough into small balls<br><br>3.Deep fry the balls in oil over medium flame until golden brown. Remove from oil & keep aside for 1 min<br><br>4.Sprinkle sugar syrup over fried Gulab Jamuns						

RASGULLA

Recipe	tsp/tbsp/ cup	g/ml	oz/lb	Ingredient	Cooked Milk protein content per 100 grams	Temperature
					2.55 grams	
**Bakes 15 medium sized Rasgulla**<br><br>**Sugar 13 grams/Rasgulla**	4 cups	1000ML	35.2oz	Whole fat pasteurized milk		Boiled: 30 minutes
	¾ cup	200 gms	8.8 oz	Sugar		
	1tsp	5gms		Sooji (semolina) or all-purpose flour or corn starch		
	2-3 tbsp	30-45ml	1.1oz-1.5oz	Lemon juice		
	Tip of knife			Cardamom powder		
	4cup	1000ml	35.2oz	Water		

Method

1. Heat milk in a heavy bottom pan
2. When the milk comes to a boil, then reduce the flame to its lowest. Add 1 to 3 tbsp lemon juice
3. Add lemon juice till the milk curdles

4. Now pour the curdled milk in the cheese cloth/muslin lined strainer or bowl
5. Drain the curdled milk using a muslin cloth. You are now left with what is known as "chena" or "paneer"
6. Squeeze the muslin cloth to remove all the water from the chena
7. Take the chena in a plate and add cornflour to it
8. Mash the chena with your hands for 10 minutes to make it soft and smooth. This is an important step to make sure that the Rasgullas are soft
9. Make small balls from the chena after the mashing is done
10. Meanwhile in a pan mix the sugar and water and let it boil at high temperature
11. Put the Rasgulla balls in the boiling sugar syrup
12. Cook the Rasgullas in sugar syrup for 18-20 minutes on high flame
13. Refrigerate the Rasgullas with the syrup and serve chilled

RAGI SARI/KANJI						
**Recipe**	**tsp/tbsp/ cup**	**g/ml**	**oz/lb**	**Ingredient**	**Cooked Milk protein content per 100 grams**	**Temperature**
					2.68 grams	
**1 Serving - 240ml**<br>**(30gms ragi sari powder)**<br><br>**Sugar 5 grams/serving**	1.5 to 2 tbsp	22 to 30 grams	1 oz	Ragi Powder		Boiled: 30 minutes
	¼ tsp	1.2 ml	0.04 oz	Ghee		
	¼ cup	62.5 ml	2.1 oz	Milk		
	½ cup	125 ml	4.4 oz	Water		
	1 tsp	5 g		Jaggery		
	¼ tsp			Cardamom powder		

Method:

1. Take 2 tbsp ragi flour in a small cup
2. Dissolve ragi flour in ½ cup water without forming any lumps. keep aside
3. Boil 1 cup water in a saucepan
4. once the water is at boiling point add in dissolved ragi flour
5. Stir continuously keeping the flame on medium
6. The mixture thickens after 5 minutes
7. Add 1 tsp jaggery
8. Stir until the jaggery dissolves completely and turns creamy
9. Now add ¼ cup milk and mix on low flame
10. Further, add ¼ tsp cardamom powder and mix well

RICE KHEER						
**Recipe**	**tsp/tbsp/ cup**	**g/ml**	**oz/lb**	**Ingredient**	**Cooked Milk Protein per 100 grams**	**Temperature**
					2.73 grams	
Serves 6<br>Sugar: 12.5 grams/serving	4cups	1000ml	33.8oz	Milk		Boiled: 30 minutes<br><br>Tip: Instead of rice it can also be made with other ingredients like vermicelli, sago
	½ cup	125gms	4.4oz	Rice		
	0.3 cup	75gms	2.6 oz	Sugar		
	10-12 nos			Raisins		
	10-12 nos			Almonds (blanched)		

Method

1.Wash and soak the rice for 30 minutes

2.Boil the soaked rice with milk in a deep pan on low flame

3.Keep stirring occasionally till the rice is cooked and milk becomes thick

4.When done add sugar, raisins and cardamoms

5.Stir till sugar gets dissolved properly

6.Transfer into a serving dish and serve

RAGI DOSA						
**Recipe**	**tsp/tbsp/ cup**	**g/ml**	**oz/lb**	**Ingredient**	**Cooked Milk protein per 100 grams**	**Temperature**
					2.92 grams	
**1serving: 5 dosas**	1cup	250gms	8.8oz	Ragi flour		Fry: 2 to 4 minutes
	1/2cup	125gms	4.4oz	Rice flour		
	1/4cup	60gms	2.1oz	Curd		
	For preparation<br><br>As needed<br>As needed			 <br>Water<br>Salt		

Method

1.Add Ragi flour, Rice flour, salt & curd to a bowl

2.Pour water as needed & make a thin batter of pouring consistency, rest aside for about 15 min

3.The batter must be running and of pouring consistency, if needed add more water to bring it to consistency

4.When the pan is hot enough then pour the batter across the pan

5. Sprinkle small amount of oil, allow to cook on a medium heat, flip and cook on the other side as well

RAVA IDLI						
**Recipe**	**tsp/tbsp/ cup**	**g/ml**	**oz/lb**	**Ingredient**	**Cooked Milk protein per 100 grams**	**Temperature**
					3.13 grams	
**1serving – 5 idlis**	1 1/4cup	300gms	10.6oz	Semolina/sooji		Steam for 15 minutes
	1cup	240ml	8.1oz	Water		
	1cup	240ml	8.1oz	Curd /yogurt		
	Tip of knife			Salt		
	For preparation<br><br>Oil for greasing idli moulds			Oil		

Method

1.Take semolina in a bowl (use the same cup to measure semolina, curd and water)

2.Then add water and curd mix it well to make thick batter

3.Cover and let the batter rest for 15 minutes

4.Take oil greased idli mould. Fill the batter till 3/4th of the mould

5.Boil water in an idli pot and keep the idli plate and steam for 15 minutes till rava idli is cooked well

6.Remove the idli and serve hot

SRIKHAND

Recipe	tsp/tbsp/ cup	g/ml	oz/lb	Ingredient	Cooked Milk protein per 100 grams	Temperature
					8.68 grams	
**Serves 4nos**<br><br>**Sugar: 7.5 grams/serving**	3cups<br>1.5 cup	720gms<br>360gms	25.3oz<br>12.6oz	Homemade curd<br>Or greek yoghurt		No cooking
	¼ cup	60gms	2.1oz	Powdered sugar		
	¼ to ½ spoon	1.2gms-2.5gms		Cardamom powder/elachi powder		
	1 pinch			Safforn strands/kesari		
	2Tbsp	60gms	2.1oz	Almonds(optional)		
	1Tbsp	30gms	1.05	Pista (optional)		

Method

1.Place a strainer over a large bowl to collect the whey

2.Spread a clean muslin or cheese cloth over the strainer

3.Add curd to the cloth. Bring all the edges together

4.Squeeze out the excess whey from the curd

5.Place the curd along with the cloth in between a thick large absorbent cloth

6.Transfer this to a plate and place a heavy object over the curd

7.Leave this in the fridge for 1 hour

8.Drained thick curd or chakka will be ready after the whey has been drained completely

9.Soak saffron in 1 tbsp hot water or hot milk

10.Powder the sugar and set aside

How to make Shirkhand

1.Remove chakka from the cloth and transfer to a mixing bowl. Most of the whey would have been drained

2.Add saffron, sugar and cardamom powder

3.Mix everything well either with a spoon or with a whisk until smooth

4.4.Garnish Shrikhand with chopped nuts

Making Shrikhand with Greek Yogurt

1.Take ½ to 1 tablespoon milk in a small bowl and warm it on a stove top or in a microwave. Add a pinch of saffron strands – about 12 to 15 strands. Mix and set aside

2.In a mixing bowl take 1.5 cups chilled Greek yogurt (about 400 grams)

3.Add sugar as per taste

4.With a wired whisk and with brisk round movements, mix the sugar with the yogurt, as well as beat the yogurt till it becomes smooth and does not have any tiny yogurt granules

5.Now add the saffron soaked milk and 1/2 teaspoon ground cardamom powder

6.Mix again very well

7.Cover the bowl with a lid and refrigerate for 30 minutes to a couple of hours

Note:

Traditional Shrikhand is made using hung curd (also known as ‘*Chakka*‘in Marathi language), a homemade yogurt that is drained of any whey.

Greek yogurt is thick unlike the usual homemade curd as the whey has been already removed during its making

Supplementary information S2: Practical guide to use milk ladder

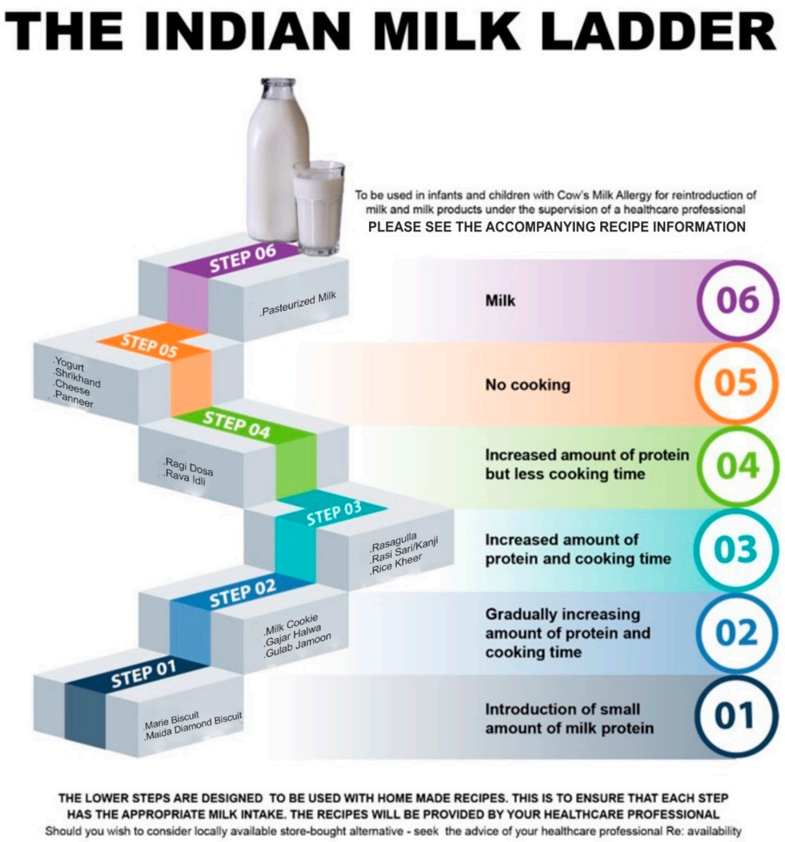

Practical Points for parents on using Indian Milk ladder at home:

- Health care practitioner (HCP) will discuss the ladder in detail with parents in detail during the clinic visit.
- Detailed recipe will be provided. (Annexure 2)
- Most children/infants will start at step one of the ladder unless they have eaten some food already; they can then move on to next step of the ladder.
- If foods on any of the steps is tolerated, child should continue to eat this (along the foods in the previous steps) and try food on the next step.
- If the child does not tolerate the food in a particular step, it is advised go back to the previous step, until further HCP guidance to step up.
- If symptoms recur it is suggested that the challenge is repeated at 6 to 8 weeks interval.
- If the food in particular step of the ladder is tolerated well, the level at which it is tolerated should be continued at, gradually increasing amounts before advancing into the next step.
- If the parents wish to give alternative food listed in the same step its recommended that they can try to increase the variety of the food in the child's diet.
- The potion details in each step to be introduced are as follows:

STEP	Food	Recommended Portion per serving
	**Step 1**	
Step 1	Marie Biscuit	½ Biscuit to start with & build up gradually
	Maida Diamond Biscuit	
	**Step 2**	
Step 2	Milk Cookie	Start with ½ Cookie & build up
	Gajar Halwa	Start with ¼ portion & increase gradually
	Gulab Jamoon	Start with ¼ portion & increase gradually
	**Step 3**	
Step 3	Rasgulla	Start with ¼ portion increase gradually
	Ragi Sari/Kanji	
	Rice Kheer	
	**Step 4**	
Step 4	Ragi dosa	Start with ¼ portion & increase gradually
	Rava idli	
	**Step 5**	
Step 5	Yoghurt	Start with ¼ portion increase gradually
	Srikhand	
	Cheese	
	Paneer	
	**Step 6**	
Step 6	Pasteurized Milk	Start with ¼ cup & increase gradually
